# Large Residual Volume, Not Low Packing Density, Is the Most Influential Risk Factor for Recanalization after Coil Embolization of Cerebral Aneurysms

**DOI:** 10.1371/journal.pone.0155062

**Published:** 2016-05-06

**Authors:** Akiyo Sadato, Motoharu Hayakawa, Kazuhide Adachi, Ichiro Nakahara, Yuichi Hirose

**Affiliations:** Department of Neurosurgery, Fujita Health University, Toyoake City, Aichi, Japan; Heinrich-Heine University, GERMANY

## Abstract

**Background:**

Tight coil packing with density of at least 20%–25% is known to be important for preventing recanalization after embolization of cerebral aneurysms. However, large aneurysms sometimes recanalize regardless of the packing density, suggesting that the absolute residual volume which is determined by aneurysm volume and packing density may be more important risk factor for recanalization. To validate this hypothesis, we analyzed the factors affecting the outcomes of treated aneurysms at our institute.

**Methods and Findings:**

We included 355 small and large aneurysms. The following six factors were obtained from every case: aneurysm volume (mL), neck size (mm), packing density (%), residual volume (mL), rupture status at presentation, and stent assistance (with or without stent). The data were then subjected to multivariate logistic regression analysis to identify significant risk factors for recanalization. Recanalization occurred in 61 aneurysms (17.2%). Significant predictors for recanalization were aneurysm volume (odds ratio, 15.3; P < 0.001) and residual volume (odds ratio, 30.9; P < 0.001), but not packing density (odds ratio, 0.98; P = 0.341). These results showed that for each 0.1-mL increase in aneurysm volume and residual volume, the risk of recanalization increased by 1.3 times and 1.4 times, respectively.

**Conclusions:**

The most influential risk factor for recanalization after coil embolization was residual volume, not packing density. The larger the aneurysm volume, the greater the packing density has to be to minimize the residual volume and risk of recanalization. Since tight coil packing has already been aimed, further innovation of coil property or embolization technique may be needed. Otherwise, different treatment modality such as flow diverter or parent artery occlusion may have to be considered.

## Introduction

Marked progress has been made in the endovascular treatment of cerebral aneurysms since the 1990s. However, recanalization continues to occur in the follow-up period after endovascular embolization. For over 10 years, it has been reported that it is important to pack coils as tightly as possible to avoid recanalization, with evidence that incomplete embolization is a major risk factor for recanalization [1–3]. A useful numerical index for evaluating the tightness of coil embolization is the packing density (PD), which is calculated as coil volume divided by aneurysm volume (AV) expressed as a percentage and low PD is reported to raise the risk of recanalization [4–10].

However, we have often seen recanalization especially in large aneurysms even with relatively high PDs. Therefore, we hypothesized that the absolute value of the residual volume (RV) of the aneurysm sac which is a composite variable of PD and aneurysm volume may be a more important risk factor for recanalization after coil embolization than the PD. This is because as the aneurysm becomes larger, so too does the absolute RV, even if the PD is equal. When there is large residual space, coils may move into the residual space resulting in coil compaction as well as the rarity of progression of thrombosis in the interstices of coil loops.

To assess the validity of our hypothesis, we retrospectively analyzed the influence of PD, RV, and other possible factors on the occurrence of aneurysm recanalization at our institute.

## Materials and Methods

### Patients

We reviewed the medical records, angiograms, and follow-up studies of 508 consecutive patients with 551 aneurysms treated by endovascular surgery at our institute between 2006 and 2012. The following cases, for which PD data and/or AV were unavailable, were excluded from the analysis: retreatments, parent artery occlusions, partially thrombosed aneurysms, and extrasaccular coils due to procedural rupture or protrusion to the parent artery. We also excluded cases without follow-up DSA or MRA from 1 year after the embolization. Three cases of giant aneurysms (>25 mm in diameter) were also excluded, because most giant aneurysms tend not to be indicated for endosaccular embolization and undergo parent artery occlusion instead. Consequently, endosaccular embolization of giant aneurysms is reserved for the very old or for prevention of re-rupture after a subarachnoid hemorrhage. Finally, 355 aneurysms were included from 341 patients.

The study was approved by the Ethics Committee of the Fujita Health University (Approval number: HM 14–125). Written informed consent for the use of their clinical records was not obtained but the study was informed in our institutional home page and opt-out policy was clarified. All the patient records/information were anonymized and de-identified prior to the analysis.

### Angiography and endovascular surgery

Diagnostic angiography was performed with a 4-French catheter before starting endovascular surgery. Aneurysm diameter, neck width, and volume were measured on volume-rendered three-dimensional angiogram images, using the default setting on a workstation (Philips Medical Systems, Best, the Netherlands). The detailed method of three-dimensional angiography and the measurements taken have been described previously [11].

A balloon-assisted technique was primarily employed for endosaccular embolization. A double-catheter technique was occasionally employed in conjunction with balloon remodeling for irregularly shaped aneurysms with multiple domes or for large aneurysms. Since September 2010, stent treatment has been available for aneurysms at our institute. Therefore, it was used in some highly selected cases, such as fusiform aneurysms and wide-necked aneurysms, for which the balloon remodeling technique was ineffective.

When selecting coils for large- or middle-sized aneurysms (diameter >7–8 mm), we opted for thicker coils (diameter 0.0135–0.015 inches) to raise PD, followed by thinner coils for filling and finishing. Small aneurysms (diameter <5–6 mm) tended to be embolized with thinner coils.

The coil volume was calculated using the equation: coil volume (V) = π[p/2]^2^L, where L represents the coil length and p represents the primary coil diameter. PD was calculated as follows: PD = [coil volume / AV] × 100%. A PD of 20% was set as the minimum requirement for satisfactory embolization, and was recalculated every time a coil was inserted. To achieve a PD that was as high as possible, coiling was continued until the microcatheter came completely out of the aneurysm.

Follow-up DSA was done 1 year after the embolization in most cases. An MRA and plain skull X-ray were performed within 1 month after treatment and every 6 months. When recanalization was suspected, DSA was done to evaluate if there was any indication for retreatment.

### Data collection and statistical analysis

Recently, some factors other than PD have been reported to be associated with recanalization and among these factors, large aneurysm size (including volume), immediate postembolization results, ruptured aneurysm, and large neck width are most often reported as significant [3,7,12–24].

The following aneurysm-related data (Table 1) were collected from the medical records together with repeat measurements from 3D-DSA images: rupture status at presentation (ruptured or unruptured), AV (mL), PD (%), postembolization RV (RV: AV [1–PD/100], mL), neck width (mm), and stent (used or not). We defined recanalization as any increased filling in the aneurysm, any change to the worse over time compared with the postoperative angiography result. Follow-up DSA was reviewed and compared directly with initial postoperative images to dichotomize the recanalization status as absent or present based on the consensus of two experienced neuroendovascular surgeons (A.S. and M.H.), without prior knowledge of the rupture status, aneurysm volume, neck width, and initial packing density.

**Table 1:** Baseline characteristics of 355 aneurysms.

	All aneurysms (N = 355)	No recanalization (N = 294)	With recanalization (N = 61)	P-value
Ruptured	39 (11.0)	27 (9.2)	12 (19.7)	0.017
Stented	28 (7.9)	23 (7.8)	5 (8.2)	0.922
Neck width (mm)	3.93 (1.77)	3.70 (1.56)	5.06 (2.27)	< 0.001
Aneurysm volume (mL)	0.199 (0.336)	0.138 (0.204)	0.492 (0.599)	< 0.001
Packing density (%)	20.97 (5.83)	21.20 (5.59)	19.86 (6.80)	0.151
Residual volume (mL)	0.159 (0.277)	0.109 (0.162)	0.400 (0.502)	< 0.001

Values are mean (SD) or count (%) for continuous or categorical variables respectively. P-value refers to no recanalization versus with recanalization comparison. Unpaired t-test, or chi-square test for continuous or categorical variables, respectively.

The aneurysm-related data (Table 1) were input as variables in stepwise multivariate regression analyses and complete regression analyses to assess the risk factors for recanalization.

Given that the AV, PD, and RV tend to be closely related, with one of the three factors being calculated from the other two, they were divided into two analyses. The first analysis included five variables, excluding RV. In the second analysis, four variables were included, with AV and PD excluded. After each stepwise logistic regression analysis, complete multivariate regression analyses were done with the remaining variables to identify their odds ratios (ORs) and 95% confidence intervals (CIs) in the most predictive logistic models. In the stepwise regression analyses, the model with the smallest AIC (Akaike information criterion) was evaluated as the most predictive model [25]. All statistical analyses were done using Systat, Version 13 (Systat Software Inc. San Jose, CA, USA), and a P-value of <0.05 was considered to indicate statistical significance.

## Results

According to the follow-up data for the 355 included aneurysms, 294 were stable and 61 (17.2%) showed recanalization. Retreatment was performed for 28 aneurysms (7.9%). The baseline characteristics of 355 aneurysms were shown in Table 1. Among the six factors, neck width, AV, and RV were significantly larger in recanalized group (t-test). Mean follow-up length was 37 ± 21 months (range 12~112 months) for all 355 aneurysms. There was no significant difference in follow-up length between the stable and recanalized group; 37 ± 21 months and 36 ± 22 months respectively. The timing of diagnosis of recanalization in 61 aneurysms was 12.2 ± 4.7 months (range 6~36, median 12 months).

In the first stepwise regression analysis with five variables excluding RV (i.e., AV, PD, rupture status, stent or not, and neck width), the most predictive regression model for recanalization included AV and rupture status but not neck width. These two factors were therefore input in the complete regression analysis. However, we also included PD because we wanted to identify the significance of PD and RV in predicting recanalization. Regression analysis (Table 2) showed that AV was a significant predictor of recanalization (OR = 15.3, 95% CI = 5.53–42.06; P < 0.001), but that PD was not (OR = 0.98, P = 0.341).

**Table 2:** Multivariate logistic regression analysis with five variables (packing density, aneurysm volume, neck, stent, and ruptured aneurysm).

Risk factors	Regression coefficient	Odds ratio	95% CI	P-value
Ruptured aneurysm	0.798	2.222	0.934–5.287	0.071
Packing density (%)	−0.025	0.975	0.926–1.027	0.341
Aneurysm volume (mL)	2.725	15.259	5.536–42.061	<0.001

In the second stepwise regression analysis of four variables (i.e., RV, rupture status, stent or not, and neck width), the most predictive regression model for recanalization included RV and rupture status. Complete regression analysis with these two factors revealed that RV was a significant predictor of recanalization (OR = 30.9, 95% CI = 0.92–5.22, P < 0.001) (Table 3). Ruptured aneurysms also had a tendency to recanalize (OR = 2.2, P = 0.07).

**Table 3:** Multivariate logistic regression analysis with four variables (residual volume, neck, stent, and ruptured aneurysm).

Risk factors	Regression coefficient	Odds ratio	95% CI	P-value
Ruptured aneurysm	0.787	2.196	8.614–110.757	0.075
Residual volume (mL)	3.43	30.887	0.924–5.221	<0.001

Finally, we calculated that for each 0.1-mL increase in the RV, the risk of recanalization increased by 1.4 times. Likewise, for each 0.1-mL increase in the AV, the risk of recanalization increased by 1.3 times. So, in the pre-embolization condition, AV is a main predictor of recanalization and then, in the post-embolization condition, the risk may be lowered by decreasing RV with high packing ratio.

## Discussion

Several reports have investigated various clinical and morphological risk factors to identify whether they are independent predictors of recanalization after embolization. However, despite the use of multivariate analyses, the results of these studies have varied. Significant factors in these reports have included large aneurysm size, aneurysm rupture, incomplete occlusion on two-dimensional postembolization angiogram, neck width, absence of a stent, low PD, posterior circulation aneurysm, and longer follow-up times [3, 7, 12–24]. Among these factors, large aneurysm size (including volume), immediate postembolization results, ruptured aneurysm, and large neck width are most often reported as significant. However, some negative reports have cast doubt over some of these dominant candidates [13, 26].

Regarding PD, there is presently no consensus as to whether it is a predictor of recanalization. Although PD has been shown to be a significant prognostic factor by univariate analysis [4–10], some negative reports exist [12, 27]. Unfortunately, majority of recent studies that have employed multivariate analyses did not include PD as a variable [3, 13, 14, 16, 17, 19–22, 24]. This is possibly because calculation of PD requires additional information that can be difficult to collect without a defect (e.g., the volume of the aneurysm and of each implanted coil). Another important reason may be that PD theoretically has a strong correlation with the postembolization results on two-dimensional angiography (complete occlusion, neck remnant, and body filling), meaning that it should be excluded from multivariate analysis.

The RV, or the absolute value of residual space of the aneurysm, has not been considered a candidate risk factor in previous studies. We chose this as a candidate because we have occasionally experienced recanalization despite a relatively high PD of over 25%, especially in large aneurysms. Therefore, we hypothesized that the RV which is a composite variable of PD and aneurysm volume may be more important than the ratio of packing alone.

In an embolization procedure, PD is a useful numeric indicator of the packing tightness. However, our study clarified that RV, and not PD, was a significant predictor of recanalization. Given that RV is a composite variable of AV and PD, the larger the aneurysm, the tighter the goal should be set for packing to achieve small RV.

Considering the mechanical properties of available coils, a certain amount of residual space may remain between coil loops, even when they are packed as tightly as possible. Some *in vitro* studies using different types of coil with silicon or glass aneurysm models have shown that maximum PD is between 30% and 55%, but that it is less than 40% in most cases [28–31]. Therefore, in large aneurysms above a certain volume, RV inevitably enlarges and makes recanalization unavoidable and different treatment modality such flow diverter or parent artery occlusion may have to be considered.

Sluzewski et al. analyzed 145 aneurysms dividing them into 3 groups; those with a volume smaller than 100 mm^3^, between 100 and 600 mm^3^, and larger than 600 mm^3^ which is corresponding to a sphere of 10.4 mm in diameter [7]. In aneurysms with the volume > 600 mm^3^, packing density was low (mean 16.7%) and coil compaction frequently occurred with more than 80% of occurrence. There was no compaction in aneurysms with a packing of 24% or more, and aneurysms with packing between 20 and 23.9% did not compact when aneurysm volume was smaller than 200 mm^3^. Thus, their finding may suggest positive correlation between aneurysm volume and minimum requirement of packing density to avoid compaction, although high packing density was hard to achieve in their large aneurysms. In our cases, recanalization occurred in some aneurysms even with a packing density of 24% or more. Among 61 recanalized aneurysms, packing density was 24% or more in 15 aneurysms, different from their cases. But our results of statistical analysis seemed to correspond with their finding that higher packing density was required to avoid recanalization in larger aneurysms.

AV and rupture status has already been identified as a significant risk factor for recanalization in previous studies [3, 7, 12, 18, 21, 22]. Our results are consistent with those findings. Our regression model suggested that the risk of recanalization increased by 1.3 times for each 0.1-mL increase in the AV. An example of 0.1-mL difference in the AV is the difference between a 9 mm sphere and 9.8 mm sphere (0.11 mL). Therefore, in large aneurysms, the risk of recanalization markedly increases as the diameter increases by 1 mm.

Although stenting has also been shown to protect against recanalization [19, 22], an absence of the protective effect has also been reported [20]. Consistent with this, our result was negative for stenting, possibly because most of the aneurysms we treated were small and treated before stents became routinely available in our country. Therefore, a relatively small number of cases were treated with stenting. Since September 2010, stents have become available for aneurysm treatment in our country; and in October 2012, our institute also changed the DSA machine to a different company’s one together with a software imaging workstation. As the method of collecting numerical data (e.g., AV and neck measurement) should be same in all aneurysms, we included all cases treated before the new DSA workstation was introduced. Therefore the cases treated with stent were limited in number (i.e., those treated between 2010 and 2012), and we recognize the need for a separate study that includes the influence of stenting in the future.

To date, reports evaluating recanalization have employed various methods to classify the degree of recanalization. Some authors have defined minor and major recanalization by the need for retreatment [3, 14, 18], while the majority of authors have considered recanalization to be any increased flow in comparison with the initial postembolization status [1, 12, 13, 17, 24]. In this study, we first tried to classify follow-up imaging into four categories: stable, minor compaction without the need for retreatment, major compaction with the need for retreatment, and recanalization with regrowth. We also tried to combine these categories in various ways to see if the differences affected the analyses, but found that the statistical analyses produced comparable results independently on the classification used (data not shown). However, because the number of aneurysms in each classification category was reduced by this process, we chose to employ a simple two-category classification—stable versus any increased flow—consistent with that used in the majority of previous studies.

The limitations of this study are its retrospective nature and the limited number of variables analyzed. In addition, only 71% of aneurysms in which all six independent variables were obtained were subjected to the analysis. This was because follow-up imaging was not always done at 1 year, especially for patients with ruptured aneurysms who were transferred to separate institutes for rehabilitation or for patients with unruptured aneurysms who were referred from distant hospitals. In addition, other location and other morphological factors were not included in this study. For location, the number of aneurysms in each location was too small to obtain significant results. Concerning the other morphological factors, we considered including the aspect ratio and dome—neck ratio, but omitted them because of their correlation with neck size, which was considered sufficient for use as a morphological factor. Different from some other studies [14, 16, 18], we found that neck size was not a significant predictor of recanalization in the multivariate analysis. However, this does not preclude the possibility that if a morphological factor is found that better reflects hemodynamic stress on the embolized aneurysm, it might show a significant influence on the rate of recanalization.

## Conclusions

RV after coil embolization which is a composite variable of PD and aneurysm volume was the most influential risk factor for recanalization. It is not adequate to set a fixed PD value as the goal of embolization. Indeed, the larger the AV, the greater the PD has to be to minimize the RV and risk of recanalization. Since tight coil packing has already been aimed universally, further innovation of coil property or embolization technique may be needed to achieve still greater PD such as coil softness, size of primary coil, and routine use of multiple catheters. Otherwise, different treatment modality such as flow diverter or parent artery occlusion may have to be considered.

## References

[1] Cognard C, Weill A, Spelle L, Piotin M, Castaings L, Rey A, et al Long-term angiographic follow-up of 169 intracranial berry aneurysms occluded with detachable coils. Radiology 1999; 212:348–356 10429689 10.1148/radiology.212.2.r99jl47348

[2] Murayama Y, Nien YL, Duckwiler G, Gobin YP, Jahan R, Frazee J, et al Guglielmi detachable coil embolization of cerebral aneurysms: 11 years' experience. J Neurosurg 2003; 98:959–966 12744354 10.3171/jns.2003.98.5.0959

[3] Raymond J, Guilbert F, Weill A, Georganos SA, Juravsky L, Lambert A, et al Long-term angiographic recurrences after selective endovascular treatment of aneurysms with detachable coils. Stroke 2003; 34:1398–1403 12775880 10.1161/01.STR.0000073841.88563.E9

[4] Uchiyama N, Kida S, Nomura M, Hasegawa M, Yamashima T, Yamashita J, et al Significance of volume embolization ratio as a predictor of recanalization on endovascular treatment of cerebral aneurysm with Guglielmi detachable coils. Interv Neuroradiol 2000; 6 Supple 1:59–63 20667222 10.1177/15910199000060S106 PMC3685936

[5] Kawanabe Y, Sadato A, Taki W, Hashimoto N. Endovascular occlusion of intracranial aneurysms with Guglielmi detachable coils: correlation between coil packing density and coil compaction. Acta Neurochir (Wien) 2001; 143:451–455 11482694 10.1007/s007010170073

[6] Tamatani S, Ito Y, Abe H, Koike T, Takeuchi S, Tanaka R. Evaluation of the stability of aneurysms after embolization using detachable coils: correlation between stability of aneurysms and embolized volume of aneurysms. AJNR Am J Neuroradiol 2002; 23:762–767 12006273 PMC7974729

[7] Sluzewski M, van Rooij WJ, Slob MJ, Bescós JO, Slump CH, Wijnalda D. Relation between aneurysm volume, packing, and compaction in 145 cerebral aneurysms treated with coils. Radiology 2004; 231:653–658 15118115 10.1148/radiol.2313030460

[8] Kai Y, Hamada J, Morioka M, Yano S, Kuratsu J. Evaluation of the stability of small ruptured aneurysms with a small neck after embolization with Guglielmi detachable coils: correlation between coil packing ratio and coil compaction. Neurosurgery 2005; 56:785–792 15792517 10.1227/01.neu.0000156790.28794.ea

[9] Yagi K, Satoh K, Satomi J, Matsubara S, Nagahiro S. Evaluation of aneurysm stability after endovascular embolization with Guglielmi detachable coils: correlation between long-term stability and volume embolization ratio. Neurol Med Chir (Tokyo) 2005; 45:561–565 16308514 10.2176/nmc.45.561

[10] Slob MJ, Sluzewski M, van Rooij WJ. The relation between packing and reopening in coiled intracranial aneurysms: a prospective study. Neuroradiology 2005; 47:942–945 16136261 10.1007/s00234-005-1446-9

[11] Sadato A, Hayakawa M, Tanaka T, Hirose Y. Comparison of cerebral aneurysm volumes as determined by digitally measured 3D rotational angiography and approximation from three diameters. Interv Neuroradiol 2011; 17:154–158 21696652 10.1177/159101991101700203 PMC3287265

[12] Piotin M, Spelle L, Mounayer C, Salles-Rezende MT, Giansante-Abud D, Vanzin-Santos R, et al Intracranial aneurysms: treatment with bare platinum coils—aneurysmpacking, complex coils, and angiographic recurrence. Radiology 2007; 243:500–508 17293572 10.1148/radiol.2431060006

[13] Grunwald IQ, Papanagiotou P, Struffert T, Politi M, Krick C, Gül G, et al Recanalization after endovascular treatment of intracerebral aneurysms. Neuroradiology 2007; 49:41–47. 17094000 10.1007/s00234-006-0153-5

[14] Ries T, Siemonsen S, Thomalla G, Grzyska U, Zeumer H, Fiehler J. Long-term follow-up of cerebral aneurysms after endovascular therapy prediction and outcome of retreatment. AJNR Am J Neuroradiol 2007; 28:1755–1761 17885238 10.3174/ajnr.A0649 PMC8134224

[15] Willinsky RA, Peltz J, da Costa L, Agid R, Farb RI, terBrugge KG. Clinical and angiographic follow-up of ruptured intracranial aneurysms treated with endovascular embolization. AJNR Am J Neuroradiol 2009; 30:1035–1040 10.3174/ajnr.A1488 19299485 PMC7051664

[16] Pierot L, Cognard C, Anxionnat R, Ricolfi F; CLARITY Investigators. Endovascular treatment of ruptured intracranial aneurysms: factors affecting midterm quality anatomic results: analysis in a prospective, multicenter series of patients (CLARITY). AJNR Am J Neuroradiol 2012; 33:1475–1480 22517279 10.3174/ajnr.A3003 PMC7966533

[17] Chalouhi N, Jabbour P, Singhal S, Drueding R, Starke RM, Dalyai RT, et al Stent-assisted coiling of intracranial aneurysms: predictors of complications, recanalization, and outcome in 508 cases. Stroke 2013; 44:1348–1353 10.1161/STROKEAHA.111.000641 23512976

[18] Lee JY, Kwon BJ, Cho YD, Kang HS, Han MH. Reappraisal of anatomic outcome scales of coiled intracranial aneurysms in the prediction of recanalization. J Korean Neurosurg Soc. 2013; 53:342–348 10.3340/jkns.2013.53.6.342 24003368 PMC3756126

[19] Chalouhi N, Tjoumakaris S, Gonzalez LF, Dumont AS, Starke RM, Hasan D, et al Coiling of large and giant aneurysms: complications and long-term results of 334 cases. AJNR Am J Neuroradiol 2014; 35:546–552 10.3174/ajnr.A3696 23945229 PMC7964717

[20] Hetts SW, Turk A, English JD, Dowd CF, Mocco J, Prestigiacomo C, et al Stent-assisted coiling versus coiling alone in unruptured intracranial aneurysms in the matrix and platinum science trial: safety, efficacy, and mid-term outcomes. AJNR Am J Neuroradiol 2014; 35:698–705 10.3174/ajnr.A3755 24184523 PMC7965822

[21] Teleb MS, Pandya DJ, Castonguay AC, Eckardt G, Sweis R, Lazzaro MA, et al Safety and predictors of aneurysm retreatment for remnant intracranial aneurysm after initial endovascular embolization. J Neurointerv Surg 2014; 6:490–494 10.1136/neurintsurg-2013-010836 23956245

[22] Murias Quintana E, Gil Garcia A, Vega Valdés P, Cuellar H, Meilán Martínez Á, Saiz Ayala A, et al Anatomical results, rebleeding and factors that affect the degree of occlusion in ruptured cerebral aneurysms after endovascular therapy. J NeuroIntervent Surg 2015; 7: 892–897 10.1136/neurintsurg-2014-011300 25358516

[23] Ogilvy CS, Chua MH, Fusco MR, Reddy AS, Thomas AJ. Stratification of recanalization for patients with endovascular treatment of intracranial aneurysms. Neurosurgery 2015; 76:390–395 10.1227/NEU.0000000000000651 25621984 PMC4637161

[24] Mascitelli JR, Oermann EK, De Leacy RA, Moyle H, Mocco J, Patel AB. Predictors of treatment failure following coil embolization of intracranial aneurysms. J Clin Neurosci 2015; 22:1275–1281 10.1016/j.jocn.2015.03.002 25986179

[25] Akaike H. Information theory and an extension of the maximum likelihood principle. Proceedings of the 2nd International Symposium on Information Theory, Petrov, B. N., and Caski, F. (eds.), Akadimiai Kiado, Budapest 1973; 267–281

[26] Gallas S, Pasco A, Cottier JP, Gabrillargues J, Drouineau J, Cognard C, et al A multicenter study of 705 ruptured intracranial aneurysms treated with Guglielmi detachable coils. AJNR Am J Neuroradiol 2005; 26:1723–1731 16091521 PMC7975138

[27] Goddard JK, Moran CJ, Cross DT, Derdeyn CP. Absent relationship between the coil-embolization ratio in small aneurysms treated with a single detachable coil and outcomes. AJNR Am J Neuroradiol 2005; 26:1916–1920 16155134 PMC8148827

[28] Piotin M, Iijima A, Wada H, Moret J. Increasing the packing of small aneurysms with complex-shaped coils: an in vitro study. AJNR Am J Neuroradiol 2003; 24:1446–1448 12917143 PMC7973684

[29] Piotin M, Liebig T, Feste CD, Spelle L, Mounayer C, Moret J. Increasing the packing of small aneurysms with soft coils: an in vitro study. Neuroradiology 2004; 46:935–939 15502997 10.1007/s00234-004-1264-5

[30] Quasar Grunwald I, Molyneux A, Kühn AL, Watson D, Byrne JV. Influence of coil geometry on intra-aneurysmal packing density: evaluation of a new primary wind technology. Vasc Endovascular Surg 2010; 44:289–293 10.1177/1538574410363916 20403951

[31] Mehra M, Hurley MC, Gounis MJ, King RM, Shaibani A, Dabus G, et al The impact of coil shape design on angiographic occlusion, packing density and coil mass uniformity in aneurysm embolization: an in vitro study. J Neurointerv Surg 2011; 3:131–136 10.1136/jnis.2010.004390 21990804

